# Skin-Only Closure Versus Bogota Bag: A Comparative Study of Complete Fascial Approximation Rates

**DOI:** 10.7759/cureus.104203

**Published:** 2026-02-24

**Authors:** Jawairia Nawaz, Wasim Ahmad, Muhammad Junaid Shah, Iram Bashir

**Affiliations:** 1 Department of General Surgery, Medical Teaching Institution Bannu (MTI) District Headquarters Teaching Hospital, Dera Ismail Khan, PAK; 2 Department of General Surgery, Gomal Medical College, Dera Ismail Khan, PAK

**Keywords:** fascial, laparostomy, loss of domain, open abdomen, surgery

## Abstract

Background: Open abdomen is a life-saving technique often used to treat serious injuries and for general surgery in emergencies. Primary fascial closure is the basic aim of open abdomen by using different techniques of closure.

Objective: To compare the frequency of achievement of primary fascial closure after skin-only closure versus a laparostomy (Bogota bag closure) among patients presenting with peritonitis or abdominal trauma at a tertiary care hospital.

Methods: A prospective cohort study was conducted at Surgical Unit D of Lady Reading Hospital, Peshawar, Pakistan, over a period of six months. A total of 220 patients with peritonitis and blunt/penetrating abdominal trauma aged 15-75 years who required damage control surgery were consecutively selected, whereas obese patients and patients managed by other techniques were excluded. Patients were grouped into group A (skin-only closure) and group B (Bogota bag closure), operated by consultants. Primary fascial closure achievement was measured during index hospitalization. Data were analyzed using Statistical Product and Service Solutions (SPSS, version 25.0; IBM SPSS Statistics for Windows, Armonk, NY).

Results: Of the 220 patients, 110 were treated with the skin-only closure technique and 110 with the Bogota bag closure technique. Primary fascial closure was achieved in 137 (62.3%) patients, out of which 89 (80.9%) patients were in group A and 48 (43.6%) patients were in group B (p<0.001).

Conclusion: The skin-only closure technique is more effective in achieving primary fascial closure as compared to the Bogota bag closure technique in patients with peritonitis and blunt/penetrating abdominal trauma.

## Introduction

Open abdomen is one of the challenging conditions for surgeons due to the physiological, metabolic, and hemodynamic implications. Open abdomen means omitting the fascial closure of the abdomen after cavity opening [1,2]. Over the years, different techniques have been developed for managing complex abdominal problems, including open abdomen and handling temporary abdominal wall closures [3]. Some clinical conditions force the surgeon to leave the abdominal cavity open after surgery, leading to an open abdomen or laparostomy [4].

Hietbrink et al. identified that most of the patients presenting with major injuries died due to intraoperative complications. Approximately 21.0% of patients died during trauma laparotomy [5]. Trauma patients presenting with hypothermia (temperature < 34°C), acidosis (pH < 7.2), persistent hypotension, and coagulopathy are at a higher risk of intraoperative complications and may require damage control surgery and/or open abdomen [6]. Early decision to use the damage control technique is also an important aspect that can decrease the rate of mortality. Therefore, trauma patients before surgery should be screened for different factors, including medical history (comorbidity), type and severity of injury, and clinical condition of the patient, along with the presence of required resources and experienced surgeons [3,6].

Secondary peritonitis or abdominal sepsis is by far the most common form of peritonitis faced in surgical practice and can occur as a result of multiple pathologies, such as a visceral perforation (e.g., perforated peptic ulcer or gall bladder), infection or inflammation (e.g., inflammatory bowel disease), and trauma, including iatrogenic injury. Most of the patients with abdominal sepsis require operative management in order to control the infectious source and eliminate microorganisms, including bacteria and toxins. The extent and severity of intra-abdominal infection determine the type of procedure [7]. A single surgery is enough to achieve it, but some cases require relook operations. Patients undergoing multiple revision surgeries usually succumb to the stress of it and may require a delay in definitive intervention until the body is healthy enough and tissue healing is adequate [8].

Patients undergoing laparotomies face many adverse ramifications, which may arise from different components of the process, including abdominal wall defects. Different temporary abdominal closure methods have been used to date, according to the disease status of the abdomen, the gap until the next surgery, and the surgeon's preference [9,10]. These methods include simple packing, skin-only closure, and Bogota bag closure. The skin-only closure technique uses the edges of the skin to contain abdominal viscera, whereas the Bogota bag closure technique employs a large intravenous bag to contain abdominal viscera [9-11].

The current study was conducted to compare the two most commonly used methods of temporary abdominal closure in Pakistan (i.e., skin-only closure and Bogota bag) to compare the achievement of primary fascial closure. Comparison of their outcomes will help a significant number of patients by preventing hernias and help surgeons in choosing the best procedure for temporary abdominal closure.

## Materials and methods

A randomized controlled trial was conducted at Surgical Unit D of Lady Reading Hospital, Peshawar, Pakistan, during a period of six months. The sample size for the study was calculated with the help of research conducted by Hu et al., who reported that the primary fascial closure was achieved in 96.4% patients in the skin-only group and in 83.3% patients in the Bogota bag group [12].

The level of significance was 5%, and the power of the test was 90%. The calculated sample size was 220 (110 in each group). Patients of both genders, ages between 15 and 75 years, who presented with peritonitis and blunt/penetrating abdominal trauma and required damage control surgery either by a skin-only closure or a Bogota bag closure technique, were selected through consecutive sampling in this study.

Patients with a body mass index (BMI) of > 30, requiring other than midline laparotomy incisions, presented with significant loss of tissue from the anterior abdominal wall, and those who did not consent to be a part of the study were excluded from this study. Peritonitis was defined as the presence of inflammation in the peritoneum because of any cause. Blunt trauma was defined as injury of the abdomen due to the application of force through any means, such as a fall from height or being hit by a blunt object. Penetrating trauma was defined as injury of the abdominal wall and viscera due to a sharp object, resulting in a peritoneal breach. Patients of all categories were assessed on medical history, clinical examination, radiological, and hematological investigations. Achievement of primary fascial closure was defined as attaining primary approximation of fascia with suture repair without any aid such as a mesh or component separation and no signs of dehiscence during hospitalization.

Study approval was obtained from the institutional review board. A written consent was also obtained from participants of the study. All selected patients included in the study were randomly allocated using random allocation software into two equal groups. The demographics of each patient were collected, followed by recording medical history and clinical examination. All the operations were performed by consultants of the Surgical Unit D (general surgeons having more than five years' experience after graduation). The primary outcome of interest was the ability to achieve primary fascial closure based on the initial closure technique during the index hospitalization. Data were interpreted through Statistical Product and Service Solutions (SPSS, version 25.0; IBM SPSS Statistics for Windows, Armonk, NY).

## Results

Of the 220 patients, 110 were treated with the skin-only closure technique and 110 with the Bogota bag closure technique during the study period. Patients have almost similar demographics, with a significant difference (p=0.006) in gender between the two groups. The majority of the patients were male (see Table 1). There was a significant difference (p<0.05) in the causes of trauma among both groups. In group A (skin-only closure), most of the patients were suffering from traumatic injuries, whereas in group B (Bogota bag closure), most of the patients were suffering from non-traumatic causes. There was also a significant difference (p<0.05) in both groups regarding the number of days since initial surgery and the number of abdominal surgeries (Table 1).

**Table 1:** Comparison of group A and group B descriptive variables (n=220)

Variables	Group A, n (%)	Group B, n (%)	Total, N (%)	P-value
Gender				
Male	89 (80.9)	71 (64.5)	160 (72.7)	0.006
Female	21 (19.1)	39 (35.5)	60 (27.3)	
Age Group				
16-30	10 (9.1)	10 (9.1)	20 (9.1)	0.024
31-45	39 (35.5)	22 (20.0)	61 (27.7)	0.0037
46-60	28 (25.5)	32 (29.1)	60 (27.3)	0.021
61-75	33 (30.0)	46 (41.8)	79 (35.9)	0.011
Cause				
Firearm Injury	25 (22.7)	6 (5.5)	31 (14.1)	0.000141
Duodenal Perforation	5 (4.5)	16 (14.5)	21 (9.5)	0.001737
Ischemic Bowel	0 (0)	5 (4.5)	5 (2.3)	
Peripancreatic Abscess	5 (4.5)	6 (5.5)	11 (5.0)	0.02338
Acute Intestinal Obstruction	10 (9.1)	18 (16.4)	28 (12.7)	0.0088
Ileal Perforation	10 (9.1)	1 (0.9)	11 (5.0)	0.0007
Intestinal Tuberculosis	5 (4.5)	6 (5.5)	11 (5.0)	0.02338
Fecal Fistula	0 (0)	11 (10.0)	11 (5.0)	
Intestinal Perforation	5 (4.5)	0 (0)	5 (2.3)	
Sigmoid Perforation	0 (0)	5 (4.5)	5 (2.3)	
Adhesion Obstruction	0 (0)	11 (10.0)	11 (5.0)	
Gangrenous Gut	0 (0)	5 (4.5)	5 (2.3)	
Perforated Gall Bladder	5 (4.5)	0 (0)	5 (2.3)	
Blunt Trauma Abdomen	12 (10.9)	0 (0)	12 (5.5)	
Mesenteric Ischemia	11 (10.0)	7 (6.4)	18 (8.2)	0.01617
Pancreatic Abscess	5 (4.5)	1 (0.9)	6 (2.7)	0.0066
Enterocutaneous Fistula	0 (0)	6 (5.5)	6 (2.7)	
Penetrating Trauma	6 (5.5)	6 (5.5)	12 (5.5)	0.0244
Perforated Duodenal Ulcer	6 (5.5)	0 (0)	6 (2.7)	
Number of Days Since Initial Surgery				
< 5	73 (66.4)	19 (17.3)	92 (41.8)	0.00001
> 5	37 (33.6)	91 (82.7)	128 (58.2)	0.000014
Number of Abdominal Surgeries				
1-3	110 (100)	87 (79.1)	197 (89.5)	0.011863
4-6	(0)	23 (20.9)	24 (10.4)	

Primary fascial closure was significantly different (p<0.05) in both groups. It was achieved in 137 (62.3%) patients, most of whom belonged to group A (Table 2).

**Table 2:** Comparison of primary fascial closure in group A and group B

Primary Fascial Closure	Group A, n (%)	Group B, n (%)	Total, n (%)	P-value
Present	89 (80.9)	48 (43.6)	137 (62.3)	0.000504
Absent	21 (19.1)	62 (56.4)	83 (37.7)	0.00013
P-values were calculated by applying chi-square test.				

Post-stratification of primary fascial closure in groups A and B shows a significant difference in gender, age in groups, and the need for surgery (Table 3).

**Table 3:** Comparison of variables with primary fascial closure in group A and group B (n=220)

Group	Group A (n=110)		Group B (n=110)		P-value
Primary Fascial Closure	Yes	No	Yes	No	
Gender					
Male	73 (82)	16 (76.2)	26 (54.2)	45 (72.6)	0.001
Female	16 (18)	5 (23.8)	22 (45.8)	17 (27.4)	0.217
Age Group					
16-30	10 (11.2)	0 (0)	4 (8.3)	6 (9.7)	0.003
31-45	37 (41.6)	2 (9.5)	10 (20.8)	12 (19.4)	<0.001
46-60	20 (22.5)	8 (38.1)	9 (18.8)	23 (37.1)	0.001
61-75	22 (24.7)	11 (52.4)	25 (52.1)	21 (33.9)	0.271
Cause					
Firearm injury	25 (28.1)	0 (0)	1 (2.1)	5 (8.1)	<0.001
Duodenal Perforation	5 (5.6)	0 (0)	3 (6.3)	13 (21)	0.001
Ischemic Bowel	0 (0)	0 (0)	2 (4.2)	3 (4.8)	-
Peripancreatic Abscess	5 (5.6)	0 (0)	1 (2.1)	5 (8.1)	0.061
Acute Intestinal Obstruction	5 (5.6)	5 (23.8)	15 (31.3)	3 (4.8)	0.06
Ileal Perforation	10 (11.2)	0 (0)	1 (2.1)	0 (0)	-
Intestinal Tuberculosis	5 (5.6)	0 (0)	2 (4.2)	4 (6.5)	0.022
Fecal Fistula	0 (0)	0 (0)	5 (10.4)	6 (9.7)	-
Intestinal Perforation	5 (5.6)	0 (0)	0 (0)	0 (0)	-
Sigmoid Perforation	0 (0)	0 (0)	2 (4.2)	3 (4.8)	-
Adhesion Obstruction	0 (0)	0 (0)	3 (6.3)	8 (12.9)	-
Gangrenous Gut	0 (0)	0 (0)	2 (4.2)	3 (4.8)	-
Perforated Gall Bladder	5 (5.6)	0 (0)	0 (0)	0 (0)	-
Blunt Trauma Abdomen	12 (13.5)	0 (0)	0 (0)	0 (0)	-
Mesenteric Ischemia	6 (6.7)	5 (23.8)	3 (6.3)	4 (6.5)	0.629
Pancreatic Abscess	0 (0)	5 (23.8)	0 (0)	1 (1.6)	-
Enterocutaneous Fistula	0 (0)	0 (0)	5 (10.4)	1 (1.6)	-
Penetrating Trauma	6 (6.7)	0 (0)	3 (6.3)	3 (4.8)	0.046
Perforated Duodenal Ulcer	0 (0)	6 (28.6)	0 (0)	0 (0)	-
Number of Days Since Initial Surgery					
< 5	68 (76.4)	5 (23.8)	8 (16.7)	11 (17.7)	<0.001
> 5	21 (23.6)	16 (76.2)	40 (83.3)	51 (82.3)	0.189
Number of Abdominal Surgeries					
1-3	89 (100)	21 (100)	36 (75)	51 (82.3)	<0.001
4-6	0 (0)	0 (0)	12 (25)	11 (17.7)	---
P-values were calculated by applying chi-square test.					

## Discussion

These procedures (skin-only closure or Bogota bag closure) are most commonly used in abdominal trauma for obtaining temporary fascial closure without the risk of complications [13]. Patients who failed to obtain primary fascial closure are at a higher risk of developing complications, prolonged hospital stay, and increased healthcare costs, and at the time of discharge from the hospital, they have a risk of worsened physical and mental well-being [14,15].

In the current study, 220 patients were selected and evaluated for primary fascial closure in group A (skin-only closure) and group B (Bogota bag closure). Out of which, 160 (72.7%) patients were male, and 60 (27.3%) were female, with a mean age of 51.63 ± 14.34 years. Demographic characteristics were similar in both groups: 89 (80.9%) vs 71 (64.5%) males and 21 (19.1%) vs 39 (35.5%) females in groups A and B, respectively. A similar study by Hu et al. conducted a retrospective analysis on trauma patients undergoing damage control surgery and reported a higher male prevalence 82.0% and a lower female prevalence 18.0% [12]. Demographic characteristics were also similar in both groups: 77.5% vs 85.0% male and 22.5% vs 15.0% female (in skin-only and Bogota bag groups, respectively). A Pakistani study by Muhammad et al. on open abdominal wounds managed by Bogota bag closure also reported a higher male prevalence 67.27% and a lower female prevalence 32.73% [16]. Reporting that abdominal trauma is more common in male patients than in female patients.

In the current study, the problems due to which patients underwent surgery were firearm injury (31, 14.1%), acute intestinal obstruction (28, 12.7%), duodenal perforation (21, 9.5%), and mesenteric ischemia (18, 8.2%). A study by Muhammad et al. reported the trauma in 69% of patients, followed by peritonitis in 18.2% and malignancy of 12.8% in patients who underwent Bogota bag closure of open abdominal wounds after exploratory laparotomy [16]. Another study by Coccolini et al. reported peritonitis in 48.7% of patients, pancreatitis in 4.2%, ischemia in 9.1%, vascular emergencies and hemorrhage in 9.4%, post-operative abdominal compartment syndrome (ACS) in 3.9%, and trauma in 20.5% [17].

In the current study, superior outcomes were achieved in terms of primary fascial closure. Previously, patients managed by primary fascial closure were reported with increased rates of ACS given the inability of the re-approximated fascia to comply with increasing visceral edema [18]. These patients suffer from increased rates of loss of abdominal domain (LOD), as well as from increased rates of mortality. However, in the current study, primary fascial closure was achieved in 137 (62.3%) patients, among whom the rate was high (89, 80.9%) in skin closure group patients and low (48, 43.6%) in Bogota bag group patients. A similar study by Hu et al. reported the primary fascial closure achieved in the skin-only group (96.4%) and the Bogota bag group (83.3%) [12]. Despite adjustments in effect modifiers and risk factors, improvement in achieving primary skin closure was great in the skin closure group as compared to the Bogota bag group.

Current research has several advantages over previously available literature; it focuses on skin-only closure and Bogota bag closure only, a study that omits selection bias, univariate directing only towards the achievement of fascial approximation, and, last but not least, it focuses on methods that are cost-effective and easily available, as our settings lack advanced methods commonly used in the developed world. With the widespread practice of damage control surgery and open abdomen comes the application of these methods and one of its most dreaded complications, the loss of domain. Similarly, current research has several limitations, including single-center research, short duration of study, and small sample size.

## Conclusions

It was concluded from the study that the frequency of achievement of primary fascial closure was high in the skin-only closure group as compared to the Bogota bag group patients. The method is easy and quick to apply in emergency settings, especially in less advanced setups lacking the facility of negative pressure wound healing systems, and saves patients from many debilitating complications such as loss of domain and ACS.
